# Language and culture in speech-language and hearing professions in South Africa: The dangers of a single story

**DOI:** 10.4102/sajcd.v65i1.594

**Published:** 2018-07-09

**Authors:** Katijah Khoza-Shangase, Munyane Mophosho

**Affiliations:** 1 Department of Speech Pathology and Audiology, School of Human and Community Development, University of the Witwatersrand, South Africa

## Abstract

**Background:**

Speech-language and hearing (SLH) professions in South Africa are facing significant challenges in the provision of clinical services to patients with communication disorders from a context that is culturally and linguistically diverse because of historic exclusions of black and African language speaking candidates in higher education training programmes. Over 20 years post the democratic dispensation, minimal changes have been noted in terms of the training, research as well as clinical service provision in these professions, although the demographic profile of students is seen to be transforming gradually.

**Objectives:**

We offer this viewpoint publication as a challenge to the professions to interrogate their academic and clinical orientation in respect of African contextual relevance and responsiveness.

**Method:**

We do this by identifying gaps within the higher education context, highlighting the influencing factors to the provision of linguistically and culturally appropriate SLH training and clinical services in South Africa, while asking questions about what SLH students and practitioners need to carefully consider.

**Results:**

We make recommendations about what needs to happen within the SLH professions in South Africa in order to stay safe from the dangers of a single story.

**Conclusion:**

We invite debate in order to allow for constructive engagement with this complex issue within the South African SLH professions.

## Speech-language and hearing professions in South Africa

Africa has and continues to experience significant health challenges, including those that affect speech-language, hearing and balance functions, which fall within the scope of practice of the speech-language and hearing (SLH) professions. Within the South African public health sector, these challenges include well-documented lack of appropriate skills, unfavourable professional-to-patient ratios with obvious incongruence between supply and demand, infrastructural constraints where access to health care services is severely compromised by limited well-functioning public health facilities, general lack of resources for the size of the population requiring health services, risk versus benefit assessments predicaments as well as challenges with translating knowledge and policies into practice for various reasons including linguistic and cultural diversity quandaries (Barratt, Khoza-Shangase, & Msimang, 2012; Khoza-Shangase, Kanji, Petrocchi-Bartal, & Farr, 2017). These challenges affect mostly the already vulnerable parts of the population such as the poor. Moreover, the research illustrates that limited rehabilitation services have been allocated to people with communication disabilities (McKenzie, 1992; Wylie, McAllister, Davidson, & Marshall, 2016).

Although one could find speech and hearing services at primary-, secondary- and tertiary-level health facilities, because of shortages of health care providers, most communities do not have the services of SLH professions in the public sector (Khoza-Shangase et al., 2017). Posts for qualified SLH professionals are also still lacking. Most SLH professions’ services are in private urban or tertiary hospitals (Makhoba & Joseph, 2016). The Health Professions Council of South Africa (HPCSA) (2017) reports that there are 572 audiologists, 1024 speech therapists and 1541 speech therapists and audiologists currently registered to provide SLH services in the country; however, these numbers do not reflect whether these professionals are in public or private health care, nor do they reflect the racial and/or linguistic profile of these professionals. This could be one of the areas that regulators such as the HPCSA need to highlight. Efforts at redress by the South African government which are aimed at equity in health are acknowledged. The implementation of the National Health Insurance (NHI) as well as the establishment of a unified health system for moving South Africa towards universal health coverage (UHC) remains silent on issues of transformation of the health care workforce, although it addresses challenges around staffing shortages.

Since the establishment of SLH professions’ training in the country, services for people with speech and hearing disabilities can be divided into those that were provided through the special education sector, and those through the health care sector. These services, regardless of the sector where provided, have been and remain unequal and insufficient across the landscape of the country, with an acknowledged lack of contextually relevant resources (Pascoe & Norman, 2011). What has improved, however, is that the Department of Health (DoH) does not provide services along racial lines anymore (van Rensburg, 2004). However, despite these new developments, press reports and academic articles report that access to health is still not equitable, especially for the rural and poverty-stricken black communities (Gilson & McIntyre, 2007; McKenzie & Müller, 2006), hence the white paper on NHI. Access to health care is also significantly impacted by language barriers. The use of English as the main language of consultation in health settings continues to create barriers for non-English speakers (Makhoba & Joseph, 2016; Pascoe, Klop, Mdlalo, & Ndhambi, 2017). This is particularly disturbing for the SLH professions where the core function of the professions is to assess and treat language and communication pathology in patients. According to the 2011 South African Speech Language and Hearing Association (SASLHA) database, most SLH professionals are in private practice (67%), in urban areas, and have a mother tongue English and/or Afrikaans and only 5% are black African language speakers. No recent statistics providing such data could be sourced; however, experientially, current authors believe that these statistics still provide a fair picture of what the current reality is. With South Africa’s demographic and linguistic profile, it is clear that the majority of black African language speakers with communication disorders do not receive health care services in their first language. This practice is contrary to what Kathard et al. (2011) advocate when they emphasise that SLH professions in South Africa need to be socially responsive and population focussed in order to make meaningful contributions to development in this country.

The current article argues for concerted efforts by the South African SLH professions to increase their attention to the influence of linguistic and cultural diversity in the provision of speech-language therapy and audiology services within this context. The current authors, who are both black Africans who speak African languages, are foregrounding this positionality in this viewpoint publication – black Africanness. In approaching this viewpoint publication, the authors were guided by Mignolo’s (2009:8) concept of epistemic disobedience which they find useful when confronting issues of exclusion, which is the current scenario for black Africans in SLH professions in South Africa. Mignolo points out that ‘the task of decolonial thinking is to affirm the epistemic right of the racially devalued’. He proposes what the current authors are calling for, a radical departure that visibilises the authors’ positionality. The current authors aim to raise the awareness of the South African SLH professions to the reality that the professions are functioning within what Mignolo (2009:8) refers to as geo-politics of knowledge, where ‘the epistemic privilege of the First World’ is the norm and where white knowledge and white history define and govern the professional culture. This is particularly important where the knowledge base used in the SLH professions excludes that of the context. The exclusion of black African indigenous knowledge, which is the current position in the country, lends the profession to the *dangers of a single story* (Adichie, 2009), where this single story creates stereotypes and beliefs about communication pathology which may be untrue or are incomplete for the South African context. Stereotypes that might present dangerous narratives about linguistic, cultural, health-seeking and health-practicing beliefs of the non-dominant, in favour of the dominant – where what the African proverb ‘Until the lion learns to write, the story will always glorify the hunter’ rings true.

Such stereotypes have significant implications for both assessment and management of patients from the ‘non-dominant culture’. This, interestingly, happens in a country where the ‘non-dominant’ culture is actually that of the dominant black majority which became ‘non-dominant’ through colonisation and apartheid, with the advent of democracy that has arguably not seen significant changes to the status quo. Although the current South African political dispensation is that of a democratic state that is governed by a black Afrocentric agenda, current authors are of the view that this remains largely at policy level and has failed to be realised at implementation (lived experience) level. The authors therefore pose specific questions that the professions should interrogate in order to swing the knowledge and clinical pendulum to be more inclusive and embrace linguistic and cultural diversity rather than tolerate it – which is arguably the current position in a country like South Africa. These questions are also aimed at questioning positions that continue to maintain the status quo.

The country has 55.6 million citizens, with black Africans accounting for over four-fifths of the population in all provinces, with the exception of Western Cape and Northern Cape (StatsSA, 2016). Furthermore, the country has 11 official languages (excluding Sign language). The last official published statistics on the linguistic profile of the country indicates that the most commonly spoken languages are isiZulu (22.7%) and isiXhosa (16%), with Afrikaans (13.5%) and English (9.6%) trailing behind, followed by Setswana (8%) and Sesotho (7.6%) (Statistics South Africa, 2011); and yet English (followed by Afrikaans) is the dominant language in the SLH professions. The South African constitution advocates for individual rights to access health services in their languages; however, this right is not protected nor enforced in practice – particularly in professions like SLH professions where speech-language pathology is the diagnosis requiring both assessment and intervention.

This article aims to highlight that language and culture in SLH assessment and therapeutic interactions play a complex role, and should not be ignored and/or superficially managed. Speech-language and hearing professions as the professions dealing with communication and communication pathology should play a centrally located role in arguing for the importance of language and culture in everyday lives, and the influence of these on health-seeking behaviours. As specialists who evaluate and treat patients with hearing, balance, speech, language, cognitive communication and swallowing disorders in individuals of all ages, from infants to the elderly, they are the professions best suited to lead the deliberations around language and culture and their influence on service provision. The current authors believe that the SLH professions can advance the argument presented in this article if they begin to *embrace diversity as a contextual strength* rather than tolerate it as a contextual complication. Within the South African context, this is in line with the South African constitution, ‘which does not tolerate diversity as a necessary evil, but affirms it as one of the primary treasures of our nation’ (Judge Langa – Constitutional Court decision in MEC for Education: KwaZulu-Natal v Pillay).

## Higher education context lacunae

It is widely acknowledged that higher education programmes in the country reflect no or slow changes in curriculum transformation to be inclusive (Seabi, Seedat, Khoza-Shangase, & Sullivan, 2014). Higher education programmes, including SLH programmes in the country, are largely still based on a Eurocentric, Western epistemology with minimal, if any, inclusion of Afrocentric epistemology and ontology. This lack of transformation of the curriculum in higher education programmes has seen recently increased calls for ‘Africanisation’ and ‘contextual relevance’ of curricula by students from the Fees Must Fall, Decolonise Curriculum student movements across the country. Furthermore, these movements have also agitated for deliberation and reviews of language policies within higher education. This language policy review call has a direct implication for South Africa’s SLH professions where the only languages that have been included in the curriculum are English and Afrikaans, with the rest of the official languages being excluded (Pascoe et al., 2017). This has also been entrenched by the fact that institutional language and culture has remained removed from that which typically represents African languages and cultures. The current authors hold a view that the existing definitions, theories, assessment and treatment strategies in SLH are narrow and hegemonic and lack diversity, contextual specificity and relevance and indigenous knowledge, which is advocated by the United Nations (UN) reports on access to health services for indigenous people (UN, 2015). The current authors’ view is further supported by Kathard and Pillay (2013) when they argue that South African SLH professions’ professional knowledge is hegemonic and limiting; and they advocate for an equity-driven population-based approach at the meso level.

Du Toit (2000) argues for there being a need to engage with the historical legacies of intellectual colonisation and racialisation as well as patriarchy. This is true for higher education as a whole, but is perhaps more urgent in the South African SLH professions where the demographic profile of the profession maintains these historical legacies which exclude certain epistemologies, with minimal cosmetic changes. It is important to note that these minimal cosmetic changes, indicated by changes in the racial composition of undergraduate student enrolments, will not ensure an epistemological evolution, which is what the current authors are calling for. The maintenance of these historical legacies, under the guise of academic freedom that academic institutions boast about, has perpetuated institutional cultures of the training programmes to lack gender, knowledge, linguistic and cultural diversity. The fact that the SLH professions in this country remain highly feminised, white, Westernised, English and mostly urban perpetuate the single story narrative which limits the significant benefits the professions could gain from embracing diversity as a contextual strength.

It is within this SLH professions context that the current authors believe in what Higher Education South Africa (HESA) (2014) puts forward about transformation when they state that:

higher education transformation entails decolonising, deracialising,^1^ demasculanising^2^ and degendering^3^ South African universities, and engaging with ontological and epistemological issues in all their complexity, including their implications for research, methodology, scholarship, learning and teaching, curriculum and pedagogy. (p. 7)

For SLH professions, the implications extend to the clinical training and consequently clinical practice in a linguistically and culturally diverse context such as South Africa. These implications are supported by evidence on global health, which indicates that groups who do not form part of the dominant culture have worse health outcomes than the dominant populations – with language as an example of a barrier to health care (Flood & Rohloff, 2018). These authors, for example, believe that indigenous languages are important for reasons of autonomy, rights, research ethics, programme efficacy and revitalisation of these languages; and they also argue that health programmes conceived and delivered using the clients’ languages are likely to be more efficacious (Flood & Rohloff, 2018). Within SLH professions, the current authors believe that actual diagnosis and treatment of language disorders cannot ethically and accurately happen without considerations of language and culture.

Currently, anecdotal evidence from graduates as well as evidence from informal appraisal of exit-level outcomes gleaned from examination papers from South African training programmes indicate that there are no set minimum standards for dealing with multilingual and multicultural societies at undergraduate level training. No minimum standards that are consistent across programmes exist for theoretical training as well as for clinical training in the influence of language and culture in SLH. The lack of diversity in the demographic profile of teaching staff and clinical educators as well as of researchers (postgraduate students) across all training programmes adds to this challenge and maintains the status quo. This has a direct implication for clinicians who practice without minimum standards for practicing in diverse societies.

These lacunae in the curriculum for training SLH professions within this context limit the facilitation and development of knowledge, attitudes and skills that are relevant and responsive to the multilingual and multicultural South African context. The fact that cultural brokers, trained interpreters as well as culturally and linguistically appropriate assessment and treatment protocols continue to be reported and evidenced as significantly lacking in this context is an indictment on the profession 23 years after South Africa achieved its democracy (Pascoe, Rogers, & Norman, 2013).

Paucity of contextually relevant published research in the area does not encourage the progression and development of the professions in this area. The limited contextually relevant published research available in this area has mostly tended to focus on translation of assessment materials, development of word lists as well as on the performance of English second-language speakers on available resources and therapists’ preparedness in managing patients from diverse cultures and languages (Barratt et al., 2012; Gonasillan, Bornman, & Harty, 2013; Khoza, Ramma, Mophosho, & Moroka, 2008; Mdlalo, Flack, & Joubert, 2016; Pascoe & Norman, 2011; Pascoe et al., 2013). In 2016, one special issue journal of the *South African Journal of Communication Disorders* (Volume 63, Number 2) was dedicated to *Language acquisition and impairment in the African languages.* As significant as this special edition issue was, it is important to problematise its list of contributors who are largely white and English or Afrikaans first-language speakers – single story narrative on display. A review of the published evidence in this only available South African profession-specific journal between 1977 and 2017 further proves the single story nature of the South African SLH professions.

The current authors, through this viewpoint publication, aim to instigate a self-reflection of the South African SLH professions by asking uncomfortable questions which, when interrogated and answered in a truthful manner, might facilitate curriculum critiquing and reviewing, research questions and methods interrogating, clinical practice reflecting as well as future directions planning for the professions. These questions are framed around what Nigerian writer Chimamanda Ngozi Adichie’s cautions about ‘the danger of a single story’ – which requires that the South African SLH professions acknowledge that the professions have and continue to present and celebrate a story that is not reflective of the diversity in the country, a story that excludes the multiple other stories that this country has to offer; and does this to the detriment of the professions’ growth and to that of efficacious clinical service provision. Current authors are of the view that biographies and national narratives of pluralities focussing on education and training, as well as clinical service provision from members of the SLH professions from diverse linguistic and cultural backgrounds within the South African context, need to be compiled and published in order to identify strengths and weaknesses of the professions as well as to archive historical data that will guide future interventions. Continued exclusion of these other narratives (black people) and the treatment of diverse languages and cultures as barriers cannot continue to be ignored. Mignolo (2009, p. 4) argues for the ‘unveiling of epistemic silences of Western epistemology and affirming the epistemic rights of the racially devalued’. In this regard, he notes that knowledge should be reframed to focus on the ‘knower’ – in this context, be inclusive of black people in the SLH professions within the South African context.

## Influencing factors to provision of linguistically and culturally appropriate speech-language and hearing services in South Africa

It is not difficult to identify factors that have an influence on progress as far as provision of culturally and linguistically appropriate SLH services in the country. The current authors have identified three main influencing factors. The first influencing factor is a challenge around student access into the training programmes that offer SLH training. Despite the acknowledged general lack of student access to, opportunity and success in higher education of black students, the SLH professions, in particular, have historically and to a large extent still currently remain exclusive to white female students. Of the gross participation rate of 17% that has been documented for the general student population, where numbers are significantly skewed to favour white students (black students 9%, mixed race students 13%, Indian students 40% and white students 70%) (CHE, 2004, 2013, 2014) – the same pattern is seen in SLH professions in the country. This unrepresentative demographic profile is seen at both undergraduate and postgraduate levels with promising changes starting to emerge at undergraduate level at some of the training programmes. This non-inclusive culture of the SLH professions at university level is also seen in participation levels at professional association levels where demographic profiles of portfolio holders within professional associations do not reflect the linguistic and cultural diversity of the professions in the country, hence the recent establishment of the National Black Speech Language Hearing Association (NABSLHA), which is seen as a clear indication of the non-inclusive nature of the current professional associations. This lack of diversity inadvertently affects the priorities that the associations engage with, their positions and stances on matters of national importance and their engagement with professional and health care challenges facing the country. This lack of diversity at training and professional levels has an influence on what the profession does and offers, and where this is performed and offered.

The second factor that the current authors believe influences SLH service delivery within a multilingual and multicultural society such as South Africa is directly linked to the undergraduate student enrolments. The limited numbers of black African students who get enrolled into the training programmes do not show good throughput rates. Anecdotal evidence as well as annual unpublished reports from the various institutions that are shared at the annual inter-university heads of departments meetings indicate that throughput, dropout and undergraduate success and graduation rates of black people in SLH professions highlight a need for improved equity of opportunity and positive outcomes for these students. This is consistent with the general findings of the higher education student population in South Africa, where, for example, in 2010, the graduation rate of black students was 16% (HESA, 2014). This factor requires careful deliberation by training programmes as access without success does not take the profession forward, and has ethical implications in a context where resources are scarce. It could also be an indication of the need for curriculum and teaching and learning strategies review to that which is not foreign to the context.

The third and arguably most important influencing factor contributing towards lack of SLH professions transformation in the country is what the authors wish to term a toxic context. This contextual toxicity consists of SLH programmes’ institutional cultures that do not welcome or embrace diversity, but rather tolerate it as a regulatory imperative. This culture of exclusion is not conducive to contextually relevant growth of the professions, and directly impacts on the access and success of those who are deemed ‘minority’ in the professions even though they are the majority in the country. The toxic institutional culture is exacerbated by the ‘academic culture’ which excludes diverse or local knowledge base that allows for academic training that is inclusive and relevant. For example, the practice where researchers exclude participants in studies for not being fluent in English, and using that as an inclusion criterion, should be carefully interrogated and its influence on research findings questioned – besides the obvious ethical implications. This, in turn, has a direct impact on ‘clinical culture’ that is strongly Western and Eurocentric in nature and positioning. This Western and Eurocentric culture was reported by Tuomi as early as in 1994 where he noted how South African SLH training reflects British and North American educational models. This therefore calls for careful considerations of providing students with epistemological access rather than just physical access as well as honest debates about which epistemologies and by whom in order to disrupt this contextual toxicity.

In order to forge a different path for the profession going forward, be it wilfully or in response to the growing discontent demonstrated by ‘fees must fall’ or ‘decolonise education’ movements, the current authors invite SLH students and practitioners to carefully and honestly engage with the following questions, and take steps to address gaps where these exist – be it by engaging in research, by mentoring, by developing tools, by engaging in curriculum developments, and so on.

## What speech-language and hearing students and practitioners need to carefully consider

For productive engagement with these questions, the current authors recommend that readers answer these questions from their own positionality and also from that of another, for example, a white clinician or educator must interrogate the questions also from the perspective of an African individual, Indian individual or a mixed race individual.

- As a student, did you see yourself in the curriculum? Did you see your client in the curriculum?
- Do you think that the research output from your profession reflects your truth? Does it reflect your world view? Does it accommodate your voice? Does it reflect the truth of the context you are in? (*knowledge base – evidence-based practice)*
- How have the dominant discourses that characterise the intellectual space in your profession developed and how have they been reproduced historically? How do they continue to be reproduced? Does the maintenance of the status quo allow for best practice in your profession?
- What are the implications of the dominant discourses for social inclusion and social justice in your professions? What are the implications of the dominant discourses for ethical and efficacious clinical care of your clients in your profession?
- What are the prevailing conceptions of epistemology^4^ and ontology^5^ and to what extent have these been or are being deracialised, degendered and decolonised in your profession?
- How do the dominant cultures in your professions affect student learning, progress and success and social equity and redress? How do the dominant cultures in your profession affect your clinical engagements and success with your patients?
- How do the dominant cultures affect research and knowledge generation in your profession?
- How do the dominant cultures also affect the development and retention of a new generation of professionals that must reflect the demographic profile of the country?
- Finally, how permeable is the current professional space in your professions to a critical reflexivity in terms of changing the status quo?

## What needs to happen within the speech-language and hearing professions in South Africa?

The current authors are of the strong view that honest acknowledgement of the serious shortcomings and problems around transformation in SLH professions in South Africa is required; and this viewpoint publication forms part of this invitation to honest engagement. Acknowledgement of discrimination and racism as one of the key features of colonialism and apartheid that continue to profoundly shape the social composition of our professions nationally is overdue. The current use of language and culture to preserve SLH professions’ whiteness needs confrontation. This reflection must also encompass deliberations around the feminised nature of our professions, and how this influences training and practice – as evidence shows that communication across gender is complex (Padavic & Reskin, 2002).

Such honest reflections will lead to greater recognition of the urgent need to re-look at and re-imagine SLH training through enhancing academic capabilities of the South African training programmes. Enhancing academic capabilities of training programmes in the form of academic support, the current authors believe, would be in line with what the CHE (2013) suggests. The CHE emphasised the importance of focussing on more than just teaching skills where tips for teaching better are provided, but should also focus on the value of meaningful reflection on contextual realities as well as thorough development of theories and scholarship on teaching and learning in this context – here the current authors refer to undergraduate curriculum reform that is of international standards but is Afrocentric. Speech-language and hearing training programmes from all South African universities have a moral obligation to actively engage with and address the legacy of apartheid in all their teaching and learning activities. Responding to this moral obligation would have a positive impact in professionals produced by these programmes, with active and visible support by professional associations or bodies that would start to reflect the demographic profile of the country in terms of composition as well as priorities – who also actively address *contextually relevant* issues within this diverse South African context.

## Conclusion

As part of transformation, the SLH professions in the country need to respond to the national calls to Africanise institutions and service delivery, which include curriculum changes to incorporate more Africa-centred courses and courses that are based on evidence that is contextually relevant and responsive; clinical care changes that are contextually relevant and responsive; research focus on local needs and issues for local benefit; clinical focus that will allow for ‘next practice’ and not just ‘best practice’; language policy that respects the fact that many people speak several languages and so teaching and learning (and clinical service provision) in only English or Afrikaans creates challenges which need addressing and so on.

In line with the goals of the South African government’s UHC through implementation of NHI, it is important that issues of diversity are addressed. If the SLH professions in the country confront this knowledge and culture hegemony, they will be seen to be in covenant with The Reconstruction and Development Programme (RDP), the Constitutional mandate based on the Section 27 of the Constitution, the 1997 White Paper for the Transformation of the Health System, as well as the Vision 2030 of the National Development Plan Vision 2030.

Transformation of the SLH professions should deal with decolonisation^6^ of the curriculum by also introducing political consciousness and critical literacy diversity as recommended by Stein (2010) where she advocates for a possession of a diversity grammar and vocabulary that facilitates a discussion of race, racism and antiracism, and parallel concepts employed in the analysis of other forms of oppression – this is performed within the regulations and scopes of the professions (National Department of Health, 2014). This is also advocated by Pillay and Kathard (2015) where they argue that if the South African SLH professions do not address the impact of imperialism, colonialism and apartheid in the curriculum; these professions will continue to provide inequitable services to the majority of the South African population. The current authors strongly argue that political consciousness in the curriculum and training will teach students how to respond to cultural context, foster connection with patients or clients and will facilitate proper and productive investments in relationships with the communities they serve. The current status quo in the way the SLH professions conduct clinical service provision with most of their patients leads to questionable efficacy and continued health inequalities, with *a culture of planisa* (Phakathi, 2006) (where the use of ad hoc interpreters is accepted as norm) that needs to be minimised, if not completely eliminated. Addressing human and language rights through policies based on social justice, human rights and equity in access to health care and rehabilitation services in SLH curricula is complex. It requires awareness and attitude shifts in training institutions, academic and clinical educators, which are pivotal to teaching about cultural competency and social inclusion to successfully prepare knowledgeable and compassionate SLH professionals. It also calls for commitment to ‘epistemic disobedience’ as conceived by Mignolo (2009). In this article, epistemic disobedience means that the South African SLH professions have to deliberately refuse to fit the script that has been crafted for them by the patriarchal, heterosexist, racist, capitalistic and colonial system; and re-imagine their own Afropolitan academic and clinical script.

‘Until the lion learns to write, the story will always glorify the hunter’ – African proverb

## Footnotes

1 Deracialisation: Attenuate or eliminate distinctive racial qualities, free from appeal to race.

2 Demasculanisation: Attenuate or eliminate distinctive masculine qualities, free from appeal to masculinity.

3 Degendering: To free from any association with or dependence on gender.

4 Epistemology: Theory of knowledge (the way we know things), the nature of knowledge and how it relates to truth, belief and justification.

5 Ontology: Nature of being (what things are), becoming, existence, or reality … what is meant by ‘being’.

6 Decolonisation: Act of getting rid of colonisation; freeing a country from being dependent on another country.
